# The Neuroendocrine Regulation of Reproductive Behavior and Emotional Control by Kisspeptin

**DOI:** 10.1210/clinem/dgaf055

**Published:** 2025-01-29

**Authors:** Edouard G Mills, Jovanna Tsoutsouki, Aureliane C S Pierret, Alexander N Comninos, Waljit S Dhillo

**Affiliations:** Section of Endocrinology and Investigative Medicine, Imperial College London, London W12 0NN, UK; Department of Endocrinology, Imperial College Healthcare NHS Trust, London W6 8RF, UK; Section of Endocrinology and Investigative Medicine, Imperial College London, London W12 0NN, UK; Section of Endocrinology and Investigative Medicine, Imperial College London, London W12 0NN, UK; Section of Endocrinology and Investigative Medicine, Imperial College London, London W12 0NN, UK; Department of Endocrinology, Imperial College Healthcare NHS Trust, London W6 8RF, UK; Section of Endocrinology and Investigative Medicine, Imperial College London, London W12 0NN, UK; Department of Endocrinology, Imperial College Healthcare NHS Trust, London W6 8RF, UK

**Keywords:** kisspeptin, KISS1, behavior, sex, emotions, mood

## Abstract

Reproductive success and ultimately species survival at a population level is contingent on a plethora of neuroendocrine signals working in concert to regulate gonadal function and reproductive behavior. Among these, the neuropeptide kisspeptin (encoded by the *KISS1/Kiss1* gene) has emerged as the master regulator of the hypothalamic-pituitary-gonadal axis. Besides the hypothalamus, both kisspeptin and its cognate receptor are extensively expressed throughout cortico-limbic brain structures in rodents and humans, which are regions traditionally implicated in behavioral and emotional responses. Thus, there exists a neuroanatomical framework through which kisspeptin can integrate reproductive behavior and emotional regulation with the reproductive axis. Accordingly, this sets the scene for recent findings derived from an assortment of species, including humans, unveiling kisspeptin as an important gatekeeper of reproductive behavior and emotional control. Herein, we summarize the major preclinical animal and human experimental evidence identifying kisspeptin as a key neuromodulator of reproductive behavior and emotional state. Such findings have laid the foundations for clinical applications of kisspeptin-based therapies for patients with related reproductive and psychosexual disorders.

Reproductive success and ultimately the perpetuation of a species is contingent on a plethora of neuroendocrine signals working in concert to regulate gonadal function and reproductive behavior. Accordingly, in most sexually reproducing species, reproductive behavior occurs when fertilization is most likely to ensue and suppressed otherwise (1). For instance, female rodents in estrus display high sexual receptivity to male mating attempts, whereas during diestrus when impregnation and reproduction are not possible, they exhibit low sexual receptivity (2, 3). Moreover, in many species, including humans, reproductive behavior is rewarding and reinforcing, presumably with the purpose of enhancing reproduction (4). On the other hand, compelling evidence reveals a marked association between mental health and reproductive disorders in women and men (5, 6). Hence, considering that reproduction and reproductive behavior and emotional regulation appear to be so tightly interconnected, it is unsurprising that they could be orchestrated by similar underlying neuroendocrine mechanisms.

To this end, the neuropeptide kisspeptin (encoded by the nonhuman *Kiss1* gene and human *KISS1* gene) has emerged as the master regulator of the reproductive axis (7). Indeed, decreased kisspeptin signaling in rodents and humans results in absent puberty and subsequent infertility (8, 9), whereas increased kisspeptin signaling results in precocious puberty (10, 11). In humans, 4 circulating isoforms exist of different amino acid lengths (kisspeptin-54, -14, -13, and -10) which are the proteolytic products of a 145-amino acid precursor polypeptide, with kisspeptin-54 as the predominant circulating form (12, 13). Each isoform shares a common C-terminal decapeptide sequence, acting as the endogenous ligand for a G-protein–coupled receptor, designated the kisspeptin receptor (*KISS1R/Kiss1r)* (14, 15).

Kisspeptin primarily stimulates the hypothalamus to regulate the hypothalamic-pituitary-gonadal (HPG) axis. Here, kisspeptin acts as an indispensable upstream regulator of gonadotropin-releasing hormone (GnRH) secretion, which in turn controls downstream gonadal function via the gonadotropins, luteinizing hormone (LH) and follicle-stimulating hormone (16, 17). Besides the hypothalamus, kisspeptin and its receptor are abundantly expressed throughout the rodent and human cortico-limbic brains (detailed below) and so expression overlaps with structures critically involved in behavioral and emotional responses. Although expression patterns do not always equate to functional roles, the extensive distribution of kisspeptin and its receptor provides a neuroanatomical framework through which kisspeptin can integrate reproductive behavior with the HPG axis. Aptly, this sets the scene for recent findings derived from animal models through to humans unveiling kisspeptin as an important gatekeeper of reproductive behavior and emotions.

In this mini-review, we summarize the major animal and human experimental evidence investigating kisspeptin as a key neuromodulator of reproductive behavior and emotional control. We also provide a summary of the expression patterns for kisspeptin and its receptor throughout the brain and associated functions where data is available. Collectively, such findings have laid important foundations for clinical applications of kisspeptin-based therapies for patients with related reproductive and psychosexual disorders.

## The Expression of Kisspeptin and Its Receptor in the Brain

### Hypothalamic Distribution

Hypothalamic kisspeptin expression varies in a species-dependent manner. In rodents, kisspeptin-expressing neurons are primarily located in 2 anatomically discrete regions: the arcuate nucleus (ARC) and the anteroventral periventricular nucleus (AVPV) (18, 19). The AVPV Kiss1 neuronal population in rodents exhibits a pronounced sexual dimorphism, with females expressing kisspeptin at levels approximately 10 times higher than males (18). In contrast, the Kiss1 neuronal population in the ARC does not display any discernible sexual dimorphism in rodents. This female-dominant kisspeptin expression highlights the critical role of AVPV Kiss1 neurons in regulating female-specific processes, including the positive feedback control of GnRH secretion and ovulation. Notably, the higher expression in females may also reflect the greater involvement of this region in regulating female sexual behavior compared to males (18), a concept explored in later sections.

In humans, the corresponding regions are the infundibular nucleus and the rostral preoptic area, respectively (20, 21). Both neuronal populations innervate GnRH neurons, but they diverge in their predominant involvement in kisspeptin-GnRH function, with ARC kisspeptin regulating GnRH pulsatility (22, 23) and AVPV kisspeptin triggering the preovulatory surge (24, 25) in females. Across a range of species, ARC kisspeptin neurons abundantly co-express the neuropeptides neurokinin-B and dynorphin, forming the KNDy (*K*isspeptin/*N*eurokinin-B/*Dy*norphin) population (rodents (26), ewes (26), mice (27), rats (28) and goats (29)), but this co-expression is less prominent in humans (30).

### Extra-Hypothalamic Distribution in Rodents

In rodents, *Kiss1* mRNA is expressed in several extra-hypothalamic regions, including the medial amygdala (MeA) and the bed nucleus of the stria terminalis (BNST) (mice (31, 32); rats (31)). The Kiss1 protein has also been identified through immunocytochemistry using the well-characterized AC566 kisspeptin antibody, in regions such as the paraventricular thalamus, locus coeruleus, and periaqueductal gray in female mice (33). Kisspeptin immunoreactive fibers have been identified in similar regions in the rat brain, including the amygdala, BNST, nucleus accumbens, caudate, and locus coeruleus (34). However, it is important to acknowledge that the detection methodology is constrained by antibody cross-reactivity with other RF amide peptides, which may impact the kisspeptin-specificity of the antibody (34).

Interestingly, *Kiss1* expression in the MeA of rodents also exhibits sexual dimorphism, which is tightly dependent on sex-steroid signaling. For instance, gonad-intact male rats exhibit higher *Kiss1* expression than gonad-intact females during diestrus. However, in females, Kiss1 expression peaks during proestrus, reaching levels comparable to those in males, coinciding with elevated sex-steroid levels (32). Consistent with this, gonadectomized male and female rodents exhibit a marked reduction in MeA *Kiss1* expression. Yet, replacement with testosterone or estrogen restores Kiss1 expression, with testosterone inducing robust increases in both sexes and estrogen similarly upregulating Kiss1 expression in both males and females (32). These findings suggest the presence of positive feedback mechanisms in the MeA in response to sex-steroid signaling. Notably, the ability of testosterone to enhance Kiss1 expression in females, and estrogen to do so in males, suggests that Kiss1 neurons in the MeA are not strictly sex-specific in their steroidal responsiveness. This non-sex-specific sensitivity might reflect the role of the MeA as a dynamic integrator of hormonal signals, facilitating sexually dimorphic behaviors, while remaining adaptable to varying hormonal environments. Indeed, while the kisspeptin-MeA population is implicated in sexual behavior in both sexes, the observed differences in Kiss1 regulation may account for its more prominent role in males (as will be discussed in later sections), where Kiss1 expression is regulated by sex-steroids without the cyclical fluctuations seen in females.

Similar to *Kiss1* expression, *Kiss1r* mRNA is widely distributed in the rodent brain, including the septum, pons, thalamus, hippocampus, amygdala, frontal cortex, striatum, dorsal cochlear, and supra-mammillary nuclei (14, 35), with the highest density in the dentate gyrus of the hippocampus (35). The presence of *Kiss1r* in these regions highlights its potential to mediate the effects of kisspeptin signaling in circuits critical for behavioral, cognitive, and emotional processes.

Additionally, neural circuits linking regions that express Kiss1/Kiss1r have been identified in rodents (discussed further in later sections). For example, in female mice, AVPV kisspeptin neurons project to a kisspeptin subpopulation within the ventromedial hypothalamus (VMH) (36), from where kisspeptin signaling modulates the key reproductive behavior lumbar lordosis (37). Moreover, Kiss1 neurons within the posterodorsal medial amygdala (MePD) communicate with the accessory olfactory bulb in both rats (38) and mice (39), suggesting a role in pheromone-mediated sexual behavior upon exposure to the scent of the opposite sex.

### Extra-Hypothalamic Distribution in Humans

In humans, *KISS1* and *KISS1R* mRNA are present in numerous cortico-limbic brain regions, including the amygdala, caudate, cingulate gyrus, globus pallidus, thalamus, hippocampus, medial frontal gyrus, nucleus accumbens, and parahippocampal gyrus (13, 15). These brain structures are integral to mood regulation, behavioral control, and reproductive behaviors (40).

Taken together, kisspeptin and its receptor are widely distributed throughout the rodent and human brains (summarized in Table 1). Their abundant expression in key brain structures implicated in behavioral and emotional control offers insights into kisspeptin's functional roles in reproductive behavior. Nonetheless, it should be noted that not all areas of kisspeptin and kisspeptin receptor expression are associated with functional roles. Indeed, these unexplored kisspeptin populations warrant future study to characterize possible functions, as has recently been ascribed to lateral septal kisspeptin cells and their involvement in sex-steroid dependent control of hypothalamic GnRH neurons (42).

**Table 1.** Extra-hypothalamic distribution of kisspeptin and kisspeptin receptors

	Mouse			Rat				Human	
	*Kiss1*		*Kiss1r*	*Kiss1*			*Kiss1r*	*KISS1*	*KISS1R*
	mRNA (31, 32)	Protein (fibers) (33, 38)	mRNA (35, 39)	mRNA (32)	Protein		mRNA (14)	mRNA (15)	mRNA (13, 15)
					(cells) (38, 41)	(fibers) (34, 38)			
Accessory olfactory bulb		+	+			+			
Amygdala	+	+		+	+	+	+	+	+
Bed nucleus of the stria terminalis	+	+			+	+			
Caudate nucleus						+		+	+
Cerebellum			+					+	+
Cingulate gyrus								+	+
Dorsal cochlear nucleus			+						
Frontal cortex							+	+	+
Globus pallidus								+	+
Hippocampus			+				+	+	+
Locus coeruleus		+				+	+	+	+
Medulla			+			+	+	+	+
Nucleus accumbens						+		+	+
Periaqueductal gray		+	+			+	+		
Pons			+			+	+		
Primary olfactory cortex							+		
Putamen								+	+
Striatum							+	+	+
Substantia nigra						+		+	+
Thalamus		+	+			+	+	+	+
Ventral tegmental area							+		

Table summarizes the brain areas outside the hypothalamus where expression of kisspeptin and its receptor has been identified in rodents and humans. “**+**” denotes confirmed expression of the kisspeptin or kisspeptin receptor mRNA or protein (cells or immunoreactive fibers)

Abbreviations: Kiss1, rodent kisspeptin expression; Kiss1r, rodent kisspeptin receptor expression; KISS1, human kisspeptin expression; KISS1R, human kisspeptin receptor expression.

## Reproductive Behavior

**Figure 1.**
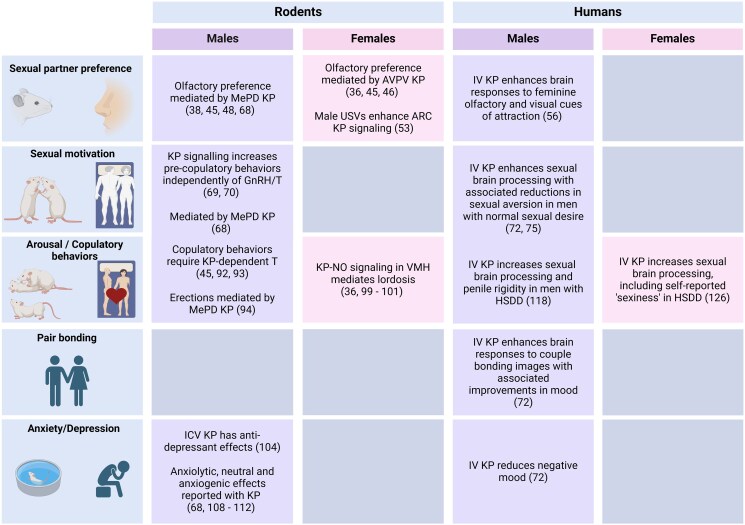
Effects of kisspeptin on reproductive behavior in rodents and humans. Figure illustrates the diverse roles of kisspeptin in modulating behaviors related to sexual partner preference, sexual motivation, arousal/copulatory behaviors, pair bonding, and mood (anxiety/depression) in male and female rodents and humans (created using BioRender.com).

Abbreviations: ARC, arcuate nucleus; AVPV, anteroventral periventricular nucleus; GnRH, gonadotropin-releasing hormone; HSDD, hypoactive sexual desire disorder; ICV, intracerebroventricular; IV, intravenous; KP, kisspeptin; MePD, posterodorsal medial amygdala; NO, nitric oxide; T, testosterone; VMH, ventromedial hypothalamus.

Reproductive behavior is a complex series of evolutionary strategies predominantly aimed at successfully producing offspring and hence species survival at a population level. It encompasses numerous key components, such as selecting and forming a bond with a potential mating partner, as well as a series of precopulatory and copulatory behaviors. Research to date implicates kisspeptin in all facets of reproductive behavior, spanning from rodents to humans, as discussed below (summarized in Fig. 1).

### Sexual Partner Preference

#### Nonhumans

Olfactory and acoustic signals provide key sensory inputs for sexual partner preference. During the precopulatory phase, sexual partners display an olfactory-driven preference for the opposite sex, and males produce ultrasonic vocalizations in response to olfactory stimulation by female pheromones.

##### Olfaction

Olfaction plays a key role in sexual partner preference through chemo-signals, termed pheromones, which are secreted by one individual and detected by conspecifics of the same species (43) and primarily processed by the accessory olfactory bulb (44). Evidence in adult male rats has identified a bidirectional neurocircuitry connecting kisspeptin neurons in the accessory olfactory bulb’s mitral cell layer to kisspeptin neuronal fibers in the MeA, which in turn project to GnRH neurons in the hypothalamic preoptic area (38). Together, this indicates that kisspeptin plays a central role in relaying pheromonal cues with the HPG axis.

To provide functional significance for the neuroanatomical connections between the olfactory system and kisspeptin populations, preclinical studies have investigated whether sex-related olfactory cues can modulate kisspeptin neuronal activity. Whereas wild-type female mice show a preference for male mice, this male-directed olfactory preference is lost in *Kiss1r* knockout (KO) mice with intact smell, even when estrogen replaced (45). Moreover, when female mice are exposed to male odors, such as urine or soiled bedding, AVPV kisspeptin *c-Fos* activity increases by almost 40% (46). Indeed, independent researchers have observed that when ovariectomized female rats (implanted with preovulatory levels of estradiol) are presented with male odors, this increased kisspeptin activity in the AVPV is associated with concomitant LH surges​ (47). Crucially, these responses are not observed in female rodents in response to female odors (36, 46).

The importance of AVPV kisspeptin neurons in modulating female rodent partner preference is further supported by separate experiments involving either *Kiss1* KO females (supplemented with estradiol and progesterone) or selective viral ablation of *Kiss1-*expressing AVPV cells, which results in loss of normal male-directed preference (36). However, male-directed preference is restored by subcutaneous kisspeptin administration in both experimental paradigms (36), demonstrating the importance of AVPV kisspeptin neurons in mediating mate preference in female mice. Mechanistic insights for the signaling pathways downstream of kisspeptin is provided by studies using transgenic GnRH-deficient female mice, which fail to display male-directed preferences, even following subcutaneous kisspeptin administration (36). Comparatively, subcutaneous GnRH administration restores male-directed preference (36), suggesting that kisspeptin signals through GnRH to regulate sexual partner preference.

Regarding male rodents, whereas wild-type mice spend over 70% of their investigatory time with female conspecifics, *Kiss1r* KO males display no preference for either sex and spend an equal amount of time investigating both sexes, even after restoring testosterone levels (45). In contrast to females, wild-type male mice show a different pattern of kisspeptin neuronal activation when exposed to opposite-sex odors. Exposure to female odors leads to a 2-fold rise in *c-Fos* positive kisspeptin neurons in the MePD and a corresponding rise in LH levels (without affecting AVPV or ARC kisspeptin activity) (48). Together, this indicates that sexual partner preference in male mice depends on MePD kisspeptin activation, whereas in females the AVPV currently has more characterized roles.

In seasonal breeders like goats and sheep, a sociosexual phenomenon known as the *ram effect* is driven by male pheromones detected by females. This effect occurs when the introduction of a male (ram/buck) into a group of anestrous females (ewes/does) overrides the normal suppressive effects of estradiol (49). Indeed, it has been demonstrated that when sexually mature males are introduced to ovariectomized goats, there is an increase in ARC kisspeptin neuronal activity, which coincides with the generation of LH pulses (50). Similarly, in anestrous ewes, exposure to a male enhances ARC kisspeptin neuronal activity and increases both LH amplitude and pulse frequency, whereas these effects are abolished by pretreatment with a kisspeptin antagonist (P-271) (51). Whether this reproductive neurohormonal response also translates into behavioral effects would be an attractive area for future investigation.

##### Audition

In rodents, in response to olfactory stimulation by female olfactory pheromones, males emit song-like ultrasonic vocalizations, which convey the male's motivational state and help attract a mate (52). It is curious that the kisspeptin receptor is expressed in the rodent dorsal cochlear nucleus (35), which may indicate kisspeptin's involvement in acoustic processing. In fact, when female mice are exposed to male ultrasonic vocalizations for 20 minutes, ARC kisspeptin neuronal activity (unlike in the AVPV) increases by up to 20%, compared to when mice are exposed to control sounds (53). This heightened kisspeptin activity positively correlates with the duration of the female's searching behavior during the first 5 minutes of exposure to male ultrasonic vocalizations, indicating that the female response to male vocalizations is linked to the activation of kisspeptin neurons (53).

Taken together, these data implicate kisspeptin signaling in relaying olfactory and auditory signals with the HPG axis in rodents (summarized in Fig. 1). However, different populations of kisspeptin neurons are activated by auditory cues in female rodents (specifically in the ARC), compared to those activated by olfactory cues (the AVPV in females and MeA in males) highlighting a complex and sexually differentiated interconnected system. Additional studies, particularly regarding kisspeptin's involvement in auditory processing (including in other species) are much warranted to characterize this further.

#### Humans

The attraction to another individual is the key initiating step for human sexual behavior, which similar to nonhuman species depends on olfactory and visual signals (54). Functional magnetic resonance imaging (fMRI) is a noninvasive approach to study kisspeptin's modulation of human attraction brain pathways. This specialized neuroimaging technique measures brain activity by detecting changes in blood flow during a specific task and so produces a brain activation map showing which regions are involved in a particular process (such as sexual behavior) (55).

To this end, the effects of acute kisspeptin-54 administration on brain processing during olfactory and facial attractiveness tasks has been investigated in 33 healthy eugonadal men (56). On exposure to a feminine scent, kisspeptin significantly enhanced brain activity in key limbic regions involved in human olfactory processing and sexual arousal (including the amygdala, caudate, putamen, and thalamus) (57-62). Regarding visual cues of attraction, kisspeptin selectively activated 2 key aesthetic brain regions, the medial prefrontal cortex and superior frontal gyrus (63-65), on viewing female faces, whereas other brain areas were unaffected (unlike in the olfactory task). This suggests that kisspeptin administration can amplify the neural aesthetic circuitry. Taken together, these findings indicate that kisspeptin enhances brain activity in response to olfactory and visual cues of attraction in men and importantly kisspeptin's effects are region-specific and dependent on the nature of the attraction cue. Crucially, and from a therapeutic perspective, the effects of kisspeptin on brain activity in this cohort of men were more pronounced in individuals with lower sexual quality of life scores, signifying the potential clinical applications of kisspeptin for patients with psychosexual disorders, as discussed later in this review.

### Sexual Motivation

Sexual motivation refers to the mechanisms that govern an individual's response to sexually relevant stimuli (66). In nonhumans, it is linked to sexual approach behaviors, whereas in humans, sexual motivation is often termed sexual desire (67).

#### Nonhumans

An important role for kisspeptin signaling in sexual motivation, specifically in the MePD, has been demonstrated in male mice using a chemogenetic approach (68). Whereas at baseline there was only a tendency for increased investigation of an estrous female over another male, selective activation of MePD kisspeptin neurons using Designer Receptors Exclusively Activated by Designer Drugs (DREADDs) doubled the amount of time spent by males with female conspecifics (68). This reveals a site-specific regulatory role for kisspeptin in the motivation to approach a sexually receptive estrous female.

Turning to evidence in rats, the effect of kisspeptin on sexual motivation and its dependence on testosterone levels has been evaluated in males following 3 interventions: intranasal administration of a GnRH analogue, intraperitoneal kisspeptin, or intranasal kisspeptin (69). During behavioral testing with an estrous female, intranasal GnRH increased circulating testosterone levels but did not affect sexual motivation. Comparatively, intraperitoneal kisspeptin enhanced both testosterone and sexual motivation, as evidenced by halving the latent time before trying to reach the female. Notably, despite not affecting testosterone levels, intranasal kisspeptin increased sexual motivation, indicating that kisspeptin is a GnRH/testosterone-independent regulator of sexual motivation in male rats in this paradigm (69).

Along similar lines, a very recent study examined kisspeptin's testosterone-independent effects in promoting other measures of precopulatory behavior in male rats (70). During the precopulatory phase of sexual interaction, males exhibit certain behaviors with receptive females, including anogenital investigation (71). In fact, peripheral kisspeptin administration to both gonadally intact and testosterone-replaced gonadectomized male rats was shown to increase the frequency of anogenital investigation of a receptive female by 2-fold compared with non-kisspeptin treated counterparts (70). Remarkably, in a third cohort of non-testosterone-replaced gonadectomized male rats, the frequency of anogenital investigation fell from weeks 2 to 5 after surgery, whereas the addition of peripheral kisspeptin treatment at week 6 restored this behavior to the same level as week 2 (70). Taken together, these findings provide further evidence for kisspeptin-mediated precopulatory behaviors in rodents, which are independent of (or at least supplemental to) testosterone.

#### Humans

In humans, the desire for sexual stimulation is an important precursor to reproduction. In 29 healthy young men, acute kisspeptin-54 administration has been shown to enhance fMRI brain activity in response to viewing sexual images (72). Specifically, kisspeptin enhanced brain activity in the anterior and posterior cingulate and amygdala, which are regions expressing kisspeptin receptors in humans (12, 13, 15) and established areas of the sexual-processing network (62, 73). Of note, kisspeptin activated key limbic brain structures more in participants with lower baseline reward-behavior scores, and the more kisspeptin enhanced activity in several limbic structures, the less aversion to sex participants exhibited, suggesting a role for kisspeptin in sexual disinhibition. It is also salient that kisspeptin administration had no effect on brain responses to other images (including negative, neutral, happy and fearful-themed images) (72). Consistent with this, in separate investigation of 27 healthy young men, acute kisspeptin-54 administration had no effect on fMRI brain responses to visual food stimuli (74). Together, this indicates that kisspeptin's effect as a neuroendocrine modulator of human brain activity is specific to the sexual-processing network and that importantly these responses correlate with reward measures and reduced sexual aversion, to ultimately drive a desire for sexual activity.

In addition to modulating task-based human brain processing, kisspeptin's effects on brain activity in the absence of a task or experimental stimuli (termed *resting state*) has been evaluated using fMRI. In 29 healthy young men, acute kisspeptin-54 administration was observed to modulate resting brain connectivity (75). Indeed, kisspeptin's modulation of a major resting-state brain network, the default mode network, was greater in men with lower baseline reward scores and correlated with subsequent increases in limbic brain activity (including the posterior cingulate and globus pallidus) on viewing sexual images, as well as reduced sexual aversion (75). These findings are particularly relevant as default mode network connectivity is known to be lower in individuals with lesser reward traits (76) and is frequently disrupted in psychosexual disorders (77). Collectively, this suggests that kisspeptin can modulate a major functionally connected resting-state network to enhance responses to sexual stimuli and reduce sexual aversion, particularly in less reward-driven individuals. This again highlights the intriguing possibility of kisspeptin-based therapies for patients with psychosexual disorders.

Moving on from fMRI, magnetic resonance spectroscopy offers a noninvasive imaging technique to quantify concentrations of neurotransmitters in the brain in vivo (78). Gamma-aminobutyric acid (GABA) is a key inhibitory neurotransmitter that underlies several common mood and behavioral disorders (79, 80) and is also known to interplay with kisspeptin signaling to modulate reproductive function (81-85). Using this neuroimaging approach, acute kisspeptin-54 administration to 19 healthy men markedly decreased central levels of GABA in the human brain (notably, in the anterior cingulate cortex) by ∼15%, an effect that was independent of downstream testosterone levels (86). Given that a comparable magnitude of GABA change has previously been reported in psychological studies with functional significance including in modulating impulsivity (79, 80), this raises the interesting possibility that kisspeptin administration could be used to modulate downstream GABA pathways to treat psychosexual and related disorders in humans.

### Pair Bonding Behavior

Pair bonding is the strong affinity that develops in some species (including humans) between individuals in a breeding pair, which is integral in driving reproduction at a behavioral level (87, 88). Unlike in nonhuman species where there is an absence of research, the body of data in humans provides valuable insights into the influence of kisspeptin in regulating pair bonding behavior (72). On viewing non-sexual couple-bonding images, acute kisspeptin-54 administration to healthy young men selectively enhanced fMRI limbic brain activity in regions associated with romantic love (globus pallidus and thalamus) (60, 89) and bonding (amygdala) (89), without affecting circulating levels of other important hormones (including oxytocin and cortisol) (72). Notably, these regions are also known to match kisspeptin receptor expression in humans (12, 13, 15). From a behavioral perspective, kisspeptin's enhanced modulation of the amygdala in response to viewing bonding images also correlated with improvements in positive mood (72). Taken together, these observations provide evidence in humans for kisspeptin's role in bonding behavior, a frequent precursor to reproduction.

### Copulatory Behavior

Copulatory behavior refers to the sequence of coordinated actions and interactions that animals engage in during mating to facilitate successful reproduction. In rodents, male copulatory behaviors involve a series of responses to a female, including mounting, thrusting, intromission, and ejaculation (90), whereas in females, the principal copulatory behavior is lumbar lordosis (91).

The role of kisspeptin in male copulatory behavior has been demonstrated using *Kiss1* and *Kiss1r* KO rodent models. Male *Kiss1r* KO mice paired with hormone-primed sexually receptive females exhibit a complete loss of copulatory behaviors (mounts, thrusts, intromissions, and ejaculation) (45). Although testosterone replacement in gonadectomized adult males rescues mounting and thrusting behaviors, ejaculation does not occur (45). This finding aligns with studies involving testosterone-replaced *Kiss1* KO male mice (92) and rats (93), which display normal copulatory behaviors except for ejaculation. Interestingly, gonadectomized adult *Kiss1* KO male rats demonstrate estrogen-induced lordosis, a behavior typical of females (93). This response is not reversed with long-term testosterone replacement initiated during puberty and maintained into adulthood but is attenuated following neonatal kisspeptin-52 (ie, the rodent equivalent of kisspeptin-54) treatment in *Kiss1* KO males (93). Therefore, kisspeptin signaling during the neonatal period may be required to establish a defeminized brain and inhibit lordosis behavior in male rats. Taken together, this series of findings suggests that kisspeptin, in conjunction with testosterone, is crucial for facilitating normal copulatory behaviors in male rodents.

Additionally, kisspeptin has a role in stimulating ex-copula erections in male rats, which are analogous to human erections in response to erotica (94). Infusion of kisspeptin into the MePD, but not into the lateral cerebro-ventricle, dose-dependently stimulated ex-copula erections, although both methods resulted in similar LH increases (94). This effect was Kiss1r-specific, as it was blocked by a Kiss1r antagonist (peptide-234) (94). This demonstrates that kisspeptin modulates ex-copula erections via the MePD independently of GnRH and LH increases. Further evidence for the importance of MeA kisspeptin neurons comes from very recent data demonstrating that 60% of kisspeptin-expressing neurons in the MePD express the GnRH receptor (GnRHR) in male mice, whereas there is no expression in the AVPV and ARC kisspeptin populations (39). From a functional perspective, using a genetic manipulation approach to inhibit GnRHR in the MePD kisspeptin neurons of male mice reduced mounting behavior, although it had no effect on intromission or ejaculation (39). This indicates that MePD kisspeptin neurons exert a significant influence driving the initiation of copulatory behavior via GnRHR in male mice.

Moving beyond rodent studies, the administration of azoxystrobin, a fungicide, to zebrafish reduced courtship behaviors, associated with reduced brain expression of *kiss1* and *kiss2* and lower peripheral kisspeptin levels (95). In bulls, lower circulating kisspeptin was associated with a longer latency from exposure to mounting female, and incomplete penile erection and protrusion, suggesting that kisspeptin levels could serve as a biomarker for sexual behavior in male domestic animals (96). In male sheep, an oral or subcutaneous DNA kisspeptin vaccine generated anti-Kiss1 antibodies, causing decreased testosterone levels and reduced sexual behaviors, thus offering an alternative to surgical castration in these animals (97, 98).

Regarding female rodents, the primary copulatory behavior is lumbar lordosis, a sexually receptive reflex characterized by dorsiflexion in response to male mounting (91). Both peripheral and intracerebroventricular kisspeptin injections induce lordosis in female mice (36, 99). In contrast, lordosis is inhibited following administration of a Kiss1r antagonist or a progesterone receptor antagonist (99). Estradiol administration intracerebroventricularly, with or without progesterone, also stimulates lordosis, but this effect is blocked by a kisspeptin antagonist (100). These findings suggest that copulatory behavior in females might be driven by sex-steroid receptor activation on kisspeptin neurons.

Furthermore, *Kiss1* KO female mice (supplemented with estradiol and progesterone) display impaired lordosis, which is rescued by peripheral kisspeptin, but not GnRH (36). Additionally, transgenic GnRH-deficient female mice display normal lordosis behavior, demonstrating that lordosis is mediated independently of GnRH-signaling (unlike the earlier discussed sexual partner preference) (36). A neuronal kisspeptin population located in the AVPV is involved in lordosis behavior as selective ablation of these kisspeptin neurons impaired lordosis, whereas their optogenetic stimulation during male mounting induced lordosis. Importantly, lordosis was rescued by a peripheral kisspeptin injection (36). Interestingly, AVPV kisspeptin neurons appear to project to other kisspeptin subpopulations in the VMH (36) and the paraventricular nucleus (37), as demonstrated by tract tracing. This is relevant, as these areas express nitric oxide synthase (nNOS), which has also been implicated in the control of reproductive behaviors. For instance, sex-steroid replaced *nNOS* KO mice exhibit attenuated lordosis, even following subcutaneous kisspeptin, whereas this is restored following co-administration of kisspeptin with a NO-donor (SNAP) (36). Conversely, when sex-steroid replaced *Kiss1* KO females receive SNAP, they display wild-type lordosis (36). When either kisspeptin or an NO-donor were directly injected into the VMH of mice, lordosis was enhanced, which did not occur when injected into the paraventricular nucleus (101). As expected, VMH administration of an nNOS inhibitor decreased lordosis (101). Overall, this indicates that kisspeptin acts via NO-signaling in the VMH to stimulate copulatory behaviors in female mice.

Collectively, in male rodents, normal copulatory behaviors in adulthood require kisspeptin-dependent stimulation of testosterone secretion, while kisspeptin signaling in the amygdala stimulates both ex-copula erections and initiation of copulatory behaviors. In female rodents, lordosis is regulated by sex-steroid stimulation of kisspeptin signaling, which acts via NO in a population of neurons in the VMH and is independent of GnRH (summarized in Fig. 1).

## Emotional Regulation

Healthy sexual functioning is a fundamental aspect of the human experience and overall well-being (102). Indeed, a parallel interaction between mood, emotions, and sexual behavior exists. It is therefore not surprising that sexual dysfunction is often implicated in depression and anxiety disorders (103). To this end, research reveals that kisspeptin signaling coordinates both sexual behavior and emotional regulation in an integrated fashion that is positive toward reproduction as discussed below (summarized in Fig. 1).

### Depression

#### Nonhumans

During a modified forced swimming test, intracerebroventricular delivery of kisspeptin to male mice dose-dependently increases climbing and swimming times, while reducing the time spent immobile, indicative of antidepressant-like effects (104). It is salient that these antidepressant-like effects were abolished by pretreatment with α2-adrenergic and 5-HT2 serotonergic receptor antagonists, suggesting that kisspeptin's antidepressant-like actions are in part mediated through the adrenergic and serotonergic systems (104). Congruent with this interaction, in male rats, chronic escitalopram treatment (a selective serotonin reuptake inhibitor [SSRI] antidepressant), results in highly elevated *Kiss1* mRNA expression in the amygdala (by 272%), and markedly increased *Kiss1r* mRNA in the hypothalamus (170%), hippocampus (117%), and cerebellum (131%) (105). Combined with kisspeptin's earlier discussed antidepressant effects (104), these findings indicate that SSRI treatment influences central kisspeptin signaling, which may explain some of their pharmacological mood-altering effects.

#### Humans

Acute kisspeptin-54 administration to healthy young men has been observed to attenuate negative mood (without affecting positive mood) (72), in keeping with the earlier preclinical animal data identifying kisspeptin's antidepressant-like actions. Regarding the brain regions putatively involved, kisspeptin administration enhanced fMRI frontal brain activity in response to viewing negative images (72). Notably, this structure is important in human negative-mood regulation (106) and expresses kisspeptin receptors in humans (12, 13, 15). Given these observations, studies of kisspeptin administration in patients with depression may prove fruitful.

### Anxiety

#### Nonhumans

Unlike the experimental evidence highlighting positive antidepressant-like effects, kisspeptin's role in modulating anxiety-like behavior remains contentious. During a novel tank diving test, intracranial administration of kisspeptin to zebrafish has been shown to double the number of top-to-bottom transitions, indicating stimulated exploratory behavior and attenuated anxiety (107). Mechanistically, this was also associated with significant increases in mRNA levels of serotonin-related genes (*pet1* and *slc6a4a*) (107). Similarly, DREADDs-induced selective stimulation of MePD kisspeptin neurons robustly increases the time male mice spent in the open arms of an elevated plus maze by 15-fold, again suggesting increased exploratory and reduced anxiety-like behaviors (68).

In contrast, other research groups have reported anxiogenic effects from kisspeptin. Intact *Kiss1r*-deleted male mice (whereby the kisspeptin receptor is rescued selectively in GnRH neurons, to ensure normal testosterone levels) have been shown to spend twice as much time in the open arms of an elevated plus maze (108). Functionally, this may indicate that kisspeptin-receptor signaling is important for anxiety related to the fear of heights. Correspondingly, in separate reports, central administration of both kisspeptin-13 (109) and kisspeptin-8 (110) to male rats decreases the time spent in the open arm of an elevated plus maze and increases corticosterone levels, suggesting anxiogenic-like kisspeptin effects.

Comparatively, other studies indicate that kisspeptin has no effect on anxiety. Central administration of kisspeptin to male rats has been observed not to affect stress-like behaviors, including grooming and locomotive parameters (111), while similarly intraperitoneal kisspeptin administration did not alter basal or stress-induced plasma corticosterone levels (112).

#### Nonhumans

To date there has been no reported effect of kisspeptin on anxiety in humans. However, given the rapid development of kisspeptin-based therapeutics, it remains important to clarify kisspeptin's effects on behavioral, biochemical, and physiological measures of anxiety in humans.

## Therapeutic Potential of Kisspeptin in Patients With Psychosexual Disorders

Sexual dysfunction encompasses a spectrum of symptoms, including lack of sexual desire, lack of sexual pleasure, failure of genital response, and orgasmic dysfunction, to name but a few (113). Of these, a persistent lack of sexual desire with marked clinical distress to the individual, termed *hypoactive sexual desire disorder* (HSDD), is one of the commonest sexual dysfunction disorders, affecting around 8% of men and 10% of women (114). Recent insights indicate that in response to sexual stimuli, those suffering with HSDD display excessive activation of higher-level cognitive brain regions (involved in self-monitoring and self-judgment), which suppresses lower-level sexual brain centers, thereby impeding normal sexual function (115-117).

Given that clinical studies in healthy volunteers demonstrate that kisspeptin robustly modulates human sexual and emotional brain processing and frequently more so in those with a lower sexual quality of life (56, 72, 74, 75, 86), subsequent studies have examined its potential to improve sexual desire in patients with HSDD (118). In a recent study of 32 eugonadal men with HSDD, in response to erotic videos, acute kisspeptin-54 administration safely and significantly deactivated brain regions involved in self-monitoring, self-judgment, and self-control (such as the bilateral parahippocampus) (119-122), while simultaneously increasing brain activity in sexual arousal centers (such as the middle frontal gyrus and anterior cingulate cortex) (62, 123-125). Crucially, kisspeptin's acute restoration of sexual brain processing induced significant increases in penile rigidity (by 56% more than placebo) and behavioral measures of sexual desire and arousal (including increased “happiness about sex” and “flushing”), providing key functional and behavioral relevance (118). Highly congruent with the earlier findings in men with normal sexual desire (56), kisspeptin's modulation of sexual brain activity was greatest in those individuals reporting higher baseline sex-related distress and lower satisfaction with sex, signifying a more pronounced therapeutic benefit for patients with the poorest sexual quality of life (118). Crucially, in addition to baseline behavioral parameters, significant correlations between kisspeptin-enhanced brain activity and behavioral measures of acute sexual function were also observed (118). For instance, on viewing erotic videos, the more kisspeptin enhanced globus pallidus activity, the more sexually “naughty” the men felt, and the more kisspeptin activated the putamen, the more “horny” and “hard” the participants felt (118). Taken together, these clinical data provide key behavioral and functional relevance for kisspeptin's enhancement of sexual brain activity by serving to strengthen feelings of sexual desire and arousal in a patient-group of men with low sexual desire.

Having established potent clinical effects in men, it is interesting to consider whether kisspeptin could offer promise for female counterparts. In a recent clinical trial of 32 eugonadal and premenopausal women with HSDD (126), in response to erotic videos, acute kisspeptin-54 administration again safely deactivated brain regions involved in self-monitoring and self-judgment (such as the inferior frontal and middle frontal gyri) (127, 128), while increasing brain activity in sexual arousal centers (such as the postcentral and supramarginal gyri) (124, 129, 130). Remarkably, this acute restoration of sexual brain activity was associated with significant increases in self-reported ratings of “feeling sexy” (126), which is important as a positive body image is a key determinant of human sexual desire and arousal (131, 132).

## Conclusions and Future Directions

Over the last 2 decades, kisspeptin has emerged as the master regulator of reproduction due to its position at the apex of the reproductive axis. Beyond the hypothalamus, both kisspeptin and its receptor are extensively distributed throughout important cortico-limbic brain regions (ie, the behavioral and emotional control centers of the brain) in rodents and humans. As detailed throughout this review, this provides a neuroanatomical framework for an expanding pool of preclinical animal evidence derived from *Kiss1* and *Kiss1r* knockout models (largely performed in combination with sex-steroid replacement to facilitate investigation of primary defects in kisspeptin signaling on reproductive behavior, rather than as a result of a hypogonadal state), as well as chemogenetic approaches and pharmacological kisspeptin administration studies. Together, these experimental models validate kisspeptin signaling as an essential neuromodulator of reproductive behavior including sexual-partner preference, sexual motivation, and copulatory behavior, while also optimizing mood and emotions in an integrated fashion that is positive toward reproduction (summarized in Fig. 1). While several of these behavior and mood-altering effects are due to direct actions of kisspeptin on its receptor in specific cortico-limbic brain regions, other effects are due to kisspeptin's unique ability to interplay and orchestrate other important neuropeptides and neurotransmitter systems residing downstream of kisspeptin neurons, such as GABA, serotonin, NO, GnRH, and of course downstream sex-steroids.

Regarding humans, clinical studies in healthy volunteers have provided valuable insights into the influence of kisspeptin in modulating sexual and emotional brain processing. Indeed, these preclinical animal and human findings have laid the foundations for recent studies in people with distressing low sexual desire, demonstrating that kisspeptin acutely and safely restores sexual brain activity, which ultimately enhances sexual desire and arousal. Given this, further human studies (including in women) are now warranted to investigate broader patient cohorts, such as other psychosexual disorders. Moreover, in order to capitalize on the clinical utility of kisspeptin-based medicines, simpler routes of administration to parenteral injection are required. Combined with the current literature, these data would provide further fundamental mechanistic and pharmacological insights for kisspeptin's importance in reproductive behavior. To this end, the available research has helped unlock kisspeptin-based therapy as an exciting, much-needed, and well-tolerated potential addition to the treatment armamentarium for managing psychosexual disorders.

## Data Availability

Data sharing is not applicable to this article as no datasets were generated or analyzed during the current study.

## Abbreviations

ARC: arcuate nucleus
AVPV: anteroventral periventricular nucleus
BNST: bed nucleus of the stria terminalis
DREADDs: Designer Receptors Exclusively Activated by Designer Drugs
fMRI: Functional magnetic resonance imaging
GABA: gamma-aminobutyric acid
GnRH: gonadotropin hormone releasing hormone
HPG: hypothalamic-pituitary-gonadal
HSDD: hypoactive sexual desire disorder
KNDy: kisspeptin, neurokinin-B, and dynorphin
KO: knockout
LH: luteinizing hormone
MeA: medial amygdala
MePD: posterodorsal medial amygdala
nNOS: nitric oxide synthase
VMH: ventromedial hypothalamus
